# Male involvement in reproductive, maternal and child health: a qualitative study of policymaker and practitioner perspectives in the Pacific

**DOI:** 10.1186/s12978-016-0184-2

**Published:** 2016-07-16

**Authors:** Jessica Davis, Joseph Vyankandondera, Stanley Luchters, David Simon, Wendy Holmes

**Affiliations:** Centre for International Health, Burnet Institute, Melbourne, Victoria Australia; Department of Epidemiology and Preventive Medicine, Faculty of Medicine Nursing and Health Science, Monash University, Victoria, Australia; International Centre for Reproductive Health, Department of Obstetrics and Gynecology, Faculty of Medicine and Health Sciences, Ghent University, Ghent, Belgium; Present Address: West Gippsland Healthcare Group, Warragul, Australia

**Keywords:** Father involvement, Men’s involvement, Male involvement, Men as partners, Expectant fathers, Maternal and child health, Pacific

## Abstract

**Background:**

The importance of involving men in reproductive, maternal and child health programs is increasingly recognised globally. In the Pacific region, most maternal and child health services do not actively engage expectant fathers and fathers of young children and few studies have been conducted on the challenges, benefits and opportunities for involving fathers. This study explores the attitudes and beliefs of maternal and child health policymakers and practitioners regarding the benefits, challenges, risks and approaches to increasing men’s involvement in maternal and child health education and clinical services in the Pacific.

**Methods:**

In-depth interviews were conducted with 17 senior maternal and child health policymakers and practitioners, including participants from five countries (Cook Island, Fiji, Papua New Guinea, Solomon Island, and Vanuatu) and four regional organisations in the Pacific. Qualitative data generated were analysed thematically.

**Results:**

Policymakers and practitioners reported that greater men’s involvement would result in a range of benefits for maternal and child health, primarily through greater access to services and interventions for women and children. Perceived challenges to greater father involvement included sociocultural norms, difficulty engaging couples before first pregnancy, the physical layout of clinics, and health worker workloads and attitudes. Participants also suggested a range of strategies for increasing men’s involvement, including engaging boys and men early in the life-cycle, in community and clinic settings, and making health services more father-friendly through changes to clinic spaces and health worker recruitment and training.

**Conclusions:**

These findings suggest that increasing men’s involvement in maternal and child health services in the Pacific will require initiatives to engage men in community and clinic settings, engage boys and men of all ages, and improve health infrastructure and service delivery to include men. Our findings also suggest that while most maternal and child health officials consulted perceived many benefits of engaging fathers, perceived challenges to doing so may prevent the development of policies that explicitly direct health providers to routinely include fathers in maternal and child health services. Pilot studies assessing feasibility and acceptability of context-appropriate strategies for engaging fathers will be useful in addressing concerns regarding challenges to engaging fathers.

## Background

The importance of involving men in reproductive, maternal and child health programs has gained increasing recognition since the mid-1990s when key international conferences in Cairo and Beijing highlighted the tremendous benefits that actively engaging men can have for the health of men, women and children [1–7]. In many contexts worldwide, men tend to be the decision makers within families and heavily influence decisions regarding contraception and STI prevention; the allocation of money, transport and time for women to attend a health centre for antenatal care or to give birth; nutrition and workload during pregnancy; and health care for children [6, 8, 9]. Yet men are often unable to make informed choices in such matters because they have been excluded from reproductive, maternal and child health services and education. Research suggests that efforts to engage men can positively influence birth spacing and use of contraceptives [10–14], maternal workload during pregnancy [15, 16], birth preparedness [17, 18], postnatal care attendance [19], and couple communication and emotional support for women during pregnancy [10, 14, 15, 20]. In addition, research into the influence of husbands and fathers on health-related behaviours suggest that building men’s knowledge regarding maternal and child health may be beneficial in terms of care-seeking for pregnancy and birth [21–31], infant feeding practices [32–36], childhood immunisation [37], and care seeking for childhood illness [24, 34, 38].

In the Pacific region, many countries have made significant progress on improving reproductive, maternal, newborn and child health in the past decade [39]. However, the burden of poor maternal and child health remains heavy. The Millennium Development Goal (MDG) Region of Oceania, which includes Pacific Island countries such as Fiji, Kiribati, Papua New Guinea (PNG), Samoa, Solomon Islands, Tonga and Vanuatu, has the second highest maternal mortality ratio by region in the world, with 187 deaths per 100,000 live births [40]. This region has an under 5 mortality rate of 51 per 1000 live births, more than double the regional MDG target for 2015 [41]. In line with growing international recognition of the important role of men in maternal and child health (MCH), some Pacific national MCH policies now highlight the importance of engaging men – particularly fathers and male partners – in health education and clinical services related to MCH [42–44]. However, literature regarding MCH service provision in the Pacific rarely mentions men or fathers, and the scant research focusing on men’s involvement in MCH suggests that, in practice, expectant fathers and fathers of young children are rarely engaged in MCH-related services [45, 46]. While several studies have explored community and health worker perceptions of male involvement in MCH in the Pacific [45–48], less is known about the views of senior MCH policymakers and practitioners (referred to here collectively as ‘MCH officials’). This paper explores the attitudes and beliefs of senior MCH officials regarding the benefits, challenges, risks and approaches to increasing men’s involvement in MCH in the Pacific region.

## Methods

Semi-structured, in-depth interviews were conducted with senior MCH policymakers and practitioners working in the Pacific, between September 2011 and March 2012. The Pacific region is defined here in line with the MDG region of Oceania [49], which includes the Cook Islands, Fiji, Kiribati, Marshall Islands, Federated States of Micronesia, Nauru, Niue, PNG, Samoa, Solomon Islands, Tokelau, Tonga, Tuvalu, and Vanuatu. The knowledge, attitudes and beliefs of senior MCH officials about men’s involvement in MCH in the Pacific are likely to influence practice in MCH services. Given the dearth of information currently available, a qualitative study design employing in-depth interviews using standard question guides was employed to build our understanding of this topic.

### Study participants

We sought to include senior MCH officials working in the Pacific region [49]. Senior MCH officials were defined as any senior policymakers working within national health departments and working on national MCH policies or plans, senior public hospital staff specialising in MCH, senior university staff involved in health worker education and training, or representatives of regional or national non-government organisations working on MCH issues. Participants were identified using ‘snowballing’ techniques: the first round of contacts (seeds) were identified by searching attendee lists of regional maternal and child health conferences and personal contacts of authors, and then by asking interviewed contacts to nominate other appropriate senior MCH policymakers and practitioners for interview.

We sent email invitations to 33 individuals to participate in this research. Of these, 18 participants responded to invitations and agreed to be interviewed, including: four senior staff members at departments of health, five participants from UN agencies (WHO and UNFPA), five senior hospital staff, two senior university staff, one from a regional NGO and one from a regional network. Participants and their organisations represented the region (4), or a specific country in the Pacific, including Cook Island (1), Fiji (1), PNG (8), Solomon Island (3) and Vanuatu (1).

### Data collection

Three interviewers conducted in-depth interviews with participants using a standard question guide. Interviews were conducted via telephone or, where possible, in person. Sixteen individuals participated in verbal interviews, and two participants providing written responses to interview questions. Of the verbal interviews, 14 interviews were conducted individually with just one participant present, while two participants from the same organisation preferred to be interviewed together. Interviews took between 30 and 60 min and addressed i) potential benefits, ii) challenges, iii) risks and iv) opportunities for increasing men’s involvement in MCH clinical and education services in the Pacific region. All interviews were conducted in English and key points and quotations recorded by hand.

### Data analysis

Interview notes were initially reviewed based on broad themes of interest, namely: potential benefits of greater involvement of fathers in MCH clinical and education services; challenges in engaging more with fathers; possible risks associated with greater father involvement in MCH; and opportunities for involving fathers in MCH. Subsequent analysis of interview notes involved inductive data-driven coding of the text to identify and synthesise recurrent issues in the data. Data was coded manually by two authors (WH and JD), and all coding reviewed by a third author (JV), with any differences in coding resolved through discussion. Relevant quotations have been used to illustrate themes in the presentation of study findings.

## Results

### Perceived benefits of greater male involvement

#### Increased access to health services for women, children and men themselves

There was wide agreement that increasing the engagement of men in MCH is important and would yield considerable health and other benefits for families. Many participants emphasised men’s cultural role in decision-making regarding access to MCH care. They highlighted the many potential benefits of giving men information to inform their decision-making about family health matters. Participants particularly highlighted the roles that men play in relation to finances and transport:

*“Men are culturally dominant. They are the head of the family, the decision makers in family planning, and decide the number of children, decide about whether to go to hospital for the baby - the breadwinner. If they see health as important, then they can provide the money for transport.”*

Those consulted commonly spoke about likely increased use of family planning, antenatal, childbirth and postnatal care services:

*“[Men] will understand more about the risks/problems that the women have to go through the reproductive life especially during the pregnancy, delivery and postnatal period and may become more supportive for seeking medical advice in [a] timely manner, decision of family spacing and contraceptive use, use of screening services …”*

Conversely, our consultations clearly identified negative outcomes associated with the current lack of male involvement:

*“Men need to be involved in decisions because we have the problem of women having poor access to care because men make all the decisions at home, then don’t allow women to come to hospital for information and care.”*

Informants also perceived greater male involvement as an important avenue for giving men information so they can support healthy behaviours and health care seeking for children, such as exclusive breastfeeding and childhood immunisation.

Several respondents noted that greater male involvement in MCH services also presents an important opportunity for health services to engage with men in order to provide both health education and services related to men’s own health:

*“Now men can focus on their own health – non-communicable diseases, weight, diet, smoking.”*

#### Increased male partner support for family planning and reproductive health services

While greater male involvement in maternal and child health need not include being present during childbirth, several informants mentioned this as a potentially life-changing event:

*“Childbirth can be a life changing experience for PNG men. I’ve heard lots of fathers talk after seeing their second or third baby born. They see and understand [their wife] differently then, are more empathetic. They are then more amenable to other sexual and reproductive health stuff.”*

Consultations also underscored the potential benefits of male involvement in childbirth on men’s understanding of the physical toll that pregnancy and childbirth takes on a woman, leading to increased understanding of the importance of longer birth intervals and smaller family size.

#### Increased uptake of STI and HIV services

Participants identified improved prevention, testing and treatment of STIs and HIV as a major benefit associated with greater male involvement. Several respondents spoke of men’s right to know about the risks of unprotected sex and STIs to the health of their partner and children, as well as to their own health. In antenatal clinics, it seems that in most settings expectant fathers are currently only contacted when a pregnant woman tests positive for an STI or HIV. This approach places responsibility on the woman and may cause harm:

*“If a woman is positive for an STI, then the husband is called to come. Either just to test or to get treatment. Usually we tell the wife to bring him… It’s difficult for them to come, there are issues of blame and conflict.”*

### Perceived challenges to increasing male involvement

#### Socio-cultural challenges

Most informants were quick to point out social and cultural factors that dissuade men from becoming more involved in MCH or dissuade women and health workers from encouraging male involvement. Informants across many countries noted that pregnancy, childbirth and care of children are seen as ‘women’s business’. They highlighted gender norms, shyness, stigma, gossip and taboos as factors that prevent men and women openly discussing matters relating to sexual and reproductive health (SRH), and men being present when groups of women gather. Contact between couples may be avoided during particular periods, such as pregnancy and immediately postpartum. Men may feel uncomfortable attending MCH clinics, due to the proximity of pregnant women and expectation that reproductive health matters will be discussed. Furthermore, women may feel uncomfortable attending antenatal care if men are present, particularly in clinics that afford little privacy or confidentiality. Some informants also voiced a concern that men may not want to attend health care services because they are "*associated with disease*".

However, there were several suggestions that informants view culture as dynamic and open to change: *“Regarding culture, it’s not how it used to be…culture is very dynamic, it’s open to new ideas.”* Some study participants noted that such traditional norms are particularly open to change when communities see clear health and other benefits:

*“Society has identified men’s roles. Traditionally these (sexual and reproductive health) were a women’s issue. But if it’s explained how they get HIV/STI and who is responsible for violence, then they realise men play an important role.”*

#### Difficulty reaching couples with health information before pregnancy

Informants noted that it is difficult to engage couples before pregnancy in order to encourage planning for pregnancy and pre-pregnancy health checks, because in many contexts couples do not come into contact with the health service or SRH information until the first pregnancy. As one informant noted: “*The antenatal clinic is the entry point to family planning. There is unfortunately no opportunity before the first pregnancy.”* This was seen to put pressure on antenatal clinics to build pregnancy and health related knowledge from a very low base. Others highlighted a woman’s desire to *“prove her fertility, to cement the relationship”* as a barrier to encouraging couples to plan pregnancies.

#### Health centre resource constraints

A large number and variety of heath systems constraints believed to mitigate against male involvement were revealed, including very under-resourced health services that do not have capacity to reach out to men. As one respondent expressed:

*“…antenatal care and clinics…are massively under-resourced and under-staffed. There is literally no space to involve men, no space on the floor, or time…Nurses and health staff are already under great stress.”*

Insufficient numbers of male health workers and inadequate training and support for male and female staff to engage men were seen as barriers to male involvement in some settings. Informants noted that insufficient training means that many health workers are not confident talking to couples together during clinic visits, particularly on sensitive topics relating to SRH. When asked whether health workers counsel couples or individuals about sex during pregnancy, several informants noted a lack of confidence among health care workers to talk about sex. One participant noted that even college trainers are not confident to talk about sex with health workers.

Inflexible clinic opening hours and men’s working time constraints were also identified as barriers. In many settings long waiting times in clinics are likely to deter men who need to return to work quickly. Many informants also mentioned that the physical layout of clinics is not “male-friendly”. They pointed to the lack of separate waiting rooms for men or couples, and large, busy waiting rooms that might be intimidating for men. Many clinics may also find it difficult to accommodate men due to lack of physical space for private consultations thus making the presence of men problematic.

#### Health worker attitudes

In some settings, participants reported that health worker attitudes may discourage men from becoming involved – "[There is the] *attitude of the service providers, especially in the older generation, that it is the women’s arena*". However, other informants stressed that *“staff are not the issue here”* and emphasised staff commitment to engaging men in health services.

#### Conceptual barriers at the policy level

Several informants spoke of major policy-level conceptual barriers to male involvement, noting that the MCH system has been designed with only women and children in mind and is therefore unable to accommodate men. There were frequent suggestions that including men would require a *“big paradigm shift”.* Although many informants saw major benefits to engaging men more in SRH and MCH, they believed that other, more fundamental, maternal and child health system issues need to be addressed first. As one informant reported: “*when provision of [antenatal care] per se is so grossly inadequate, then male involvement is just not a priority.”*

### Potential risks associated with greater male involvement

In general there was limited discussion of risks associated with male involvement. But several informants noted concerns about putting extra stress on health workers and limited resources: *“clinics simply won’t cope.”* Other concerns included unintentionally dissuading single women from attending clinics alone when couple attendance is encouraged, and men taking even greater control over women’s domains. There were also some concerns voiced about efforts to raise awareness among men of the importance of STI prevention and treatment and MCH, when services are not available to meet increases in demand: *“Awareness without services is a waste of money.”*

### Opportunities for greater male involvement

#### Father interest in participation in MCH

Most informants expressed the view that many fathers would be interested to participate in MCH services if they were invited to do so, and that in some situations, some fathers have already begun to participate in MCH services during pregnancy and birth:

*“They enjoy it, find it fascinating. At [a private hospital] the husbands come to the antenatal clinic. They learn how to support their wife. They get to cut the cord, and to hold baby first. Even ‘bushy men’ think its great.”*

Several participants suggested that men feel ‘left out’, that *“programs are all about women and children and no one is looking after [men]”* and that men feel “*like a forgotten and blamed bunch”.* In relation to family planning, some participants spoke of men seeking care despite feelings of shame and embarrassment: “*Often the men come at night. They hide themselves and come when it’s dark to our house.”* There was also recognition that for men to attend health services requires bravery: “*Occasionally a brave guy stays [in the clinic] with his wife.”* Informants also highlighted a sense of responsibility among some men – “*people understand they have a responsibility” –* which could be leveraged to engage men in MCH issues.

#### Culturally appropriate messaging

Several participants argued that messages that build on traditional cultural roles and values are more likely to be successful, and that activities should be *“culturally sensitive, but spur changes”.* Such messages could speak to men’s traditional role as provider, with several participants noting that men may easily be convinced to better participate if they were taught about the economic benefits or health benefits associated with preventative care and male involvement in MCH.

#### Engaging boys and men throughout the life-cycle

Many informants underscored the need to involve men early in the life-cycle. Participants generally agreed that both young men and women should be educated in SRH and MCH, for example through school programs “*from the very beginning”,* but acknowledged potential opposition by some faith-based organisations. Despite such difficulties, informants in several countries noted ongoing efforts to improve SRH education in school settings, primarily through ‘family life education’ or ‘life skills education’ curricula. One respondent noted that it is *“really critical to create the language of responsibility*” among young people and that young men in particular are *“not understanding their role as a responsible father”.* Pre-marriage counselling through community structures was suggested by several respondents, but the high incidence of pregnancy before marriage and unplanned pregnancy were noted as limitations of this strategy.

#### Engaging institutions

The need to work through a variety of institutions was also noted. Many mentioned the need to engage with community structures, such as the church, community leaders, and schools. Participants spoke of the importance of engaging with politicians and local leaders to ensure they are informed and political will is mobilised. Participants also noted the need to engage a range of sectors, including education, labour or employment, as well as health, in order to spur change.

#### Engaging men in both community and clinical settings

It is clear that our informants thought in terms of a variety of settings where men might receive information and services relevant to SRH and MCH. Outreach by health workers and peer education were supported, with greater emphasis on peer education. Strategies that reached men in ‘male spaces’, such as during work breaks, betel nut or ‘grog sessions’ were suggested. In places where men’s clinics are available, group talks on issues relevant to MCH were suggested. Other spaces included church groups, schools, workplaces, and in other community gatherings. Some mentioned a need for workplace policies to enable greater male involvement in SRH and MCH, such as allowing men time to attend MCH services. Other suggested strategies for reaching men included mass-media strategies such as talk-back radio and TV.

#### Father-friendly clinics

Policymakers and practitioners outlined a variety of ways that health services could be made more ‘male-friendly’, including providing waiting spaces where men would feel comfortable, such as a separate room where men or couples can wait. Ensuring that staff have the time to engage men and women effectively was highlighted as an issue. The consultations also revealed the importance of making male health workers available and training all health workers to *“include men in all discussions when they come with their pregnant partners”*. Some respondents noted that simply having male staff in clinics can challenge the belief that clinics are "*women’s areas*".

Most participants supported the concept of a routine couple antenatal visit because it’s important for *“men to be there to hear this for themselves”*, while noting that men currently rarely or never attend an antenatal care consultation with their pregnant partner. Many recognised that pregnancy is a time when expectant fathers are more receptive to information about MCH, when “*their ears are open.”* When asked which visit would be the most appropriate visit for the couple visit, most suggested the first visit or ‘booking visit’. This is because detection of health problems and counselling occur at this and it gives men the earliest opportunity to support their wives. Some suggested both the first and last (fourth) antenatal visit, as the last visit is an opportunity for health staff to review and remind both parents about plans for birth. However, it was clear that many respondents had not thought seriously about routine couples visits previously and had not had the opportunity to think through the details of such a visit. Respondents warned that a couple visit should not be compulsory as this might prevent single, unaccompanied women attending ANC.

It was clear that men often accompany their wives to the antenatal clinic although they are rarely included in the consultation: *“If they bring the husband, he is usually hanging around outside*”. There was also some recognition that this represents a potential opportunity for contact with expectant fathers. For example, one participant had tried providing group talks to men and women about the topic of sex during pregnancy, to which *“Any men in the car park would come and listen.”*

## Discussion

Understanding the views of senior MCH policymakers and practitioners is important in designing and implementing context-specific, appropriate strategies to increase male involvement in maternal and child health in the Pacific. This research revealed strong agreement amongst policymakers regarding the benefits of involving men in MCH, including increased use of clinic services by women and children, increased use of family planning, and allowing men to support practices that promote MCH and challenge those behaviours that are detrimental to MCH. Such findings are in keeping with international findings regarding the greater decision-making power [24, 50] and low health knowledge of men [3, 24, 50], and their greater openness to new information about their role as husband and father during significant life events such as pregnancy and the birth of a child [47, 51, 52].

Participants in this study also reported beliefs that including men in MCH services may have benefits for men’s own health. In many settings, men have very little contact with the formal health system, particularly for preventative services, and prefer to seek curative services from a traditional healer or pharmacy [53]. For men, as for women, pregnancy and early childhood provides an opportunity to link parents to the health system. Efforts to engage men in MCH education and clinical services should therefore seize opportunities to provide men with information and services related to their own health. Health workers and educators providing men or couples with information on health during pregnancy and postpartum should routinely provide men with information about healthy behaviours to reduce the risk of both communicable and non-communicable diseases. Men accompanying their female partner to antenatal or postnatal clinics should also be offered testing and treatment for STIs and other infections. In all cases, men should routinely be provided with information on men’s health services available locally.

While all MCH officials participating in this research described clear benefits of greater male involvement in MCH in terms of health outcomes for women and children, many participants identified substantial barriers to engaging fathers in MCH education and clinical services. Barriers to father participation in MCH highlighted by MCH official in this research, including the belief that it is inappropriate for men to actively participate or take an active interest in MCH, and men feeling embarrassed or uncomfortable attending clinical services with their partner or child, are similar to findings from male involvement research with community members and health workers elsewhere in the world [37, 47, 50, 52, 54–61]. Importantly, participants in this study tended to conceptualise interventions to engage men in MCH as requiring men to attend clinical services with their female partner. However, promising findings from the international literature, that may be applied in Pacific contexts, suggest that men can be encouraged to take a more active, positive role in MCH using alternative strategies such as men-only group talks or one-on-one peer-education [10, 62–65], community meetings [66–68], distribution of information, education and communication materials [13, 16], or mass-media campaigns [18, 64, 65, 67, 69, 70]. These types of community-based interventions, as well as school-based programs, may also be appropriate in Pacific contexts in which many first pregnancies are unplanned and where first pregnancies are often the first point of health education for couples, a challenge highlighted in this study.

Despite these sociocultural challenges to male involvement in MCH, MCH officials consulted in this study tended to report beliefs that many men in the Pacific would welcome greater involvement in MCH education and clinical services. This finding is in line with the results of other qualitative research in Laos [52], South Africa [71], Uganda [72], and PNG [47]. In other contexts where MCH education and services are considered ‘women’s business’, simply inviting male partners to attend antenatal clinics, via a written letter, has been effective in making men feel more welcome and increasing couple attendance [73], particularly when invitations are tailored to local health concerns [74]. Our findings that the attitudes of some staff, inadequate numbers of male staff and lack of training for all staff on how to engage men in MCH, also suggest that engaging men needs to feature in health worker recruitment and training. While the attitudes and capacity of health workers has been identified as a barrier to male involvement in other research [45, 47, 50, 52, 58], training and support to all health workers, and recruiting male staff, can facilitate engagement of men in maternal and child health [4, 6, 59, 75]. In clinics, providing waiting areas and consultation spaces that men feel comfortable in, or separate spaces for men, was recommended by many of our participants and has proven effective in other contexts [76, 77]. Changes such as providing a separate entrance or waiting area for men, or displaying posters, magazines or educational DVDs that target men can make clinics less daunting and more educational for men.

Some of the changes suggested here to make clinics more ‘father-friendly’ require health service providers to have a more welcoming attitude towards fathers attending the clinic and to be mindful of the needs of fathers, but will require minimal additional resources. Other initiatives, such as changes to recruitment and training of health workers or changes to clinic infrastructure are likely to have more substantial resource implications. Considerations of resource constraints is particularly important given that many participants in this study expressed concerns regarding the ability of already over-stretched health staff and infrastructure to cater to expectant or new fathers, a perceived barrier found elsewhere [50]. Health service providers are unlikely to embrace new approaches, regardless of effectiveness, if they present an added burden that exceeds capacity. These findings underscore the need for holistic approaches to men’s involvement in MCH, that build awareness regarding the benefits of engaging fathers in MCH, while also building health system capacity to engage and serve men.

Substantial variations in social, cultural, policy and resource environments across the Pacific mean that there is unlikely to be one approach to engaging men in MCH appropriate to all communities or countries. Rather, a range of strategies is required. While Pacific actors can draw on the global evidence regarding effective approaches to engaging men in MCH, our finding suggest that concerted, policy-level efforts to increase men’s engagement in MCH clinical and education services is unlikely to occur in this region, until Pacific-specific, context- and resource-appropriate strategies for engaging men have been pilot-tested and proven feasible, acceptable and effective. Several MCH officials participating in this research expressed support for program strategies that work within or build upon cultural norms that support maternal and child health. Building on cultural norms such as men’s role in caring for their family, can be an effective strategy for encouraging improved health behaviours. For example, research shows that men socialised to be the providers and protectors of the family can be encouraged to share decision-making more equitably with their female partner when the benefits of doing so to the health of their families are clear [11, 13, 14, 17]. Furthermore, if supported adequately, many men will challenge traditional practices that might endanger their partner’s health [71, 78]. Importantly, a systematic review of interventions to improve gender-based inequality and equity in health conducted by WHO found that programs that seek to address gender-inequalities that lead to poor health outcomes are often more successful than those that simply accommodate or work around gender inequalities [67]. In Pacific contexts, initiatives that work with both men and women in examining prevailing gender norms and roles and the impact these have on health, while also engaging men to play a positive role in supporting the health of their female partners and children, may therefore be most effective in improving MCH outcomes. In most settings, initiatives to address underlying gender-inequalities that lead to poor health are unlikely to be implemented through the formal health system, requiring partnership with non-government organisations working to improve MCH and gender equality.

When asked about potential risks associated with involving men more in MCH, some participants spoke of the potential for some men to use their involvement in MCH services to exert control over choices and information usually controlled by women, a concern highlighted elsewhere in the world [6, 67, 79]. Additional risks of including men in clinical services include women feeling less free to discuss confidential information with health workers, the risk of violence or divorce when men learn information about their partners’ STI, HIV, contraceptive or other health status, or unintentionally dissuading women from attending services when they cannot bring a male partner [53, 79–82]. Programs that seek to increase men’s engagement with MCH should therefore explicitly delineate men’s rights to information and services verses women’s rights to privacy and autonomy. Men have a right to information and services that will affect their own health and that will enable them to avoid behaviors that may pose a risk to the health of their partners and children. However, men should not automatically be allowed to participate in maternal health consultations or be given access to the personal health information of their female partner, unless their partner consents to this. Efforts to engage men in MCH – whether through community-based or clinical services – ​should also carefully avoid unintentionally giving the impression that men should be the sole decision-maker regarding issues related to MCH. Findings of this study and the international literature underscore the need to involve women in program design, to pilot test communication materials and strategies, and explicitly promote equitable couple communication and decision-making for health [6, 67]. Programs to engage men in MCH clinical services must also allow women to choose how and when male partners are present and involved in maternal health clinical services. Health workers providing MCH care should routinely ask women if they would like their male partner to join the consultation, or in the case of antenatal care, give pregnant women an invitation that they have the option of passing on to their male partner to attend subsequent antenatal visits.

This study has some important limitations. Only 17 out of 33 people invited to participate did so, while the remainder of invitees did not respond to our invitation. This may be a source of selection bias because those self-selecting to participate may be more supportive of male involvement in MCH than those who declined to participate. Non-respondents did not give reasons for not participating, therefore we are unable to further examine this possible source of selection bias. Use of ‘snowballing’ as a selection strategy may have led to bias because participants might be more likely to recommend additional participants that they know are supportive of engaging men in MCH. Finally, the researchers have previously advocated publically for greater health service engagement with fathers and this may have influenced both participant recruitment and induced participants to provide socially desirable responses.

### Further research and dissemination

Most senior MCH policymakers and practitioners participating in this research articulated a range of benefits that would result from greater male involvement in MCH. This suggests that evidence-based, context-specific strategies that have been pilot-tested for feasibility and acceptability are likely to be well received by senior MCH officials and that, provided these strategies are resource-appropriate, advocacy to increase men’s engagement with MCH education and clinical services may gain traction at least at the senior levels. A search of the published literature reveals no rigorously evaluated male involvement intervention in this region and no quantitative studies of the health benefits of male involvement for mothers and babies. These findings indicate that (1) better dissemination of known impacts of male involvement is needed and (2) rigorously evaluated Pacific-specific male involvement pilot projects or trials which measure the impact on health outcomes may be valuable in encouraging action at the policy level. Policymakers and planners consulted in this research consistently highlighted the importance of locally appropriate strategies for increasing male involvement, designed on a strong understanding of local cultural and social norms. Program design is therefore likely to benefit from sound formative research into the knowledge, attitudes and practices of local communities and health workers, and from formative research to test the feasibility of strategies prior to implementation.

## Conclusion

Although our study suggested that senior MCH policymakers and practitioners in the Pacific perceive many benefits from engaging fathers in MCH in terms of health outcomes for women and children, substantial challenges exist in engaging fathers. Perceived barriers to engaging fathers in MCH-related education and clinical service may prevent the development of policies that explicitly direct health service providers to include fathers in MCH services routinely. Pilot studies that assess the feasibility and acceptability of Pacific-specific, context-appropriate strategies to increase father involvement will be useful in addressing policymakers concerns regarding barriers to engaging fathers. Our findings suggest that efforts to increase father involvement in MCH services in the Pacific will require initiatives to engage boys and men of all ages in MCH in both community and clinic settings, and to engage both men and women in addressing gender inequalities that lead to poor health. It will also be necessary to improve MCH infrastructure and service delivery to include expectant fathers. Pilot studies should therefore focus on identifying appropriate and effective strategies in these identified areas.

## Abbreviations

MCH, maternal and child health; PNG, Papua New Guinea; SRH, sexual and reproductive health; STI, sexually transmitted infection
